# Supplementary material for: Let’s talk about PFAS: Inconsistent public awareness about PFAS and its sources in the United States
Source: PLoS One. 2023 Nov 16;18(11):e0294134. doi: 10.1371/journal.pone.0294134 (PMC10653490; doi:10.1371/journal.pone.0294134)
Supplement: S1 Table — (PDF) [file pone.0294134.s001.pdf]

Table S1. Unadjusted demographic characteristics of all survey respondents.

Characteristic	n (%)*
**Sex/Gender**	
Male	529 (48%)
Female	565 (51%)
Other	4 (0.4%)
No Answer	2 (0.2%)
**Age**	
18:24	125 (11%)
25:34	192 (17%)
35:44	204 (19%)
45:54	198 (18%)
55:64	171 (16%)
65+	208 (19%)
No Answer	2 (0.2%)
**Race/Ethnicity**	
American Indian/Native American or Alaska Native	15 (1.4%)
Asian	49 (4.5%)
Hispanic or Latino or Spanish Origin of any race	109 (9.9%)
Black or African American	119 (11%)
Native Hawaiian or Other Pacific Islander	3 (0.3%)
White or Caucasian	723 (66%)
Other	21 (1.9%)
Two or More	57 (5.2%)
No Answer	4 (0.4%)
**Educational Attainment**	
Some high school	47 (4.3%)
High school or GED	418 (38%)
Associate degree	178 (16%)
Bachelor's degree	246 (22%)
Master's degree	132 (12%)
Doctorate or terminal degree	28 (2.5%)
Other	40 (3.6%)
No answer	11 (1.0%)

*N = 1,100
