# Supplementary material for: Let’s talk about PFAS: Inconsistent public awareness about PFAS and its sources in the United States
Source: PLoS One. 2023 Nov 16;18(11):e0294134. doi: 10.1371/journal.pone.0294134 (PMC10653490; doi:10.1371/journal.pone.0294134)
Supplement: S2 Table — (PDF) [file pone.0294134.s002.pdf]

Table S2. Questions and response options used in survey instrument.

Questions	Response options
**What is your main source of drinking water?**	☐ Unfiltered tap water<br>☐ Filtered tap water<br>☐ Bottled/prepackaged water<br>☐ Other
**To your knowledge, has your primary source of drinking water been impacted by PFAS?**	☐ Yes<br>☐ No
**How concerned are you about PFAS being in your drinking water?**	☐ Not at all concerned<br>☐ Slightly concerned<br>☐ Moderately concerned<br>☐ Very concerned<br>☐ Extremely concerned
**To your knowledge, has your community been exposed to PFAS?**	☐ Yes<br>☐ No<br>☐ Not sure
**How would you describe your knowledge about PFAS as an environmental contaminant?**	☐ I've heard of it, and don't know what it is<br>☐ I've heard of it or seen it somewhere, but don't know what it is<br>☐ I think I know what it is<br>☐ I'm confident I know what it is
**What percentage of the U.S. population do you think has been exposed to PFAS?**	Scale, 0-100
* **How familiar are you with the following items as potential sources of PFAS contamination?**<br>Drinking water<br>Public waterways near waste disposal sites<br>Soils near waste disposal sites<br>Dairy products<br>Fresh produce<br>Freshwater fish<br>Seafood<br>Food packaging<br>Non-stick cookware<br>Personal hygiene products<br>Household products (fabrics, cleaning products, paints, and sealants)<br>Fire extinguishing foams<br>Fertilizers from wastewater treatment plants	☐ Not at all familiar<br>☐ Slightly familiar<br>☐ Moderately familiar<br>☐ Very familiar<br>☐ Extremely familiar

*** Please rate your intention to change your use of the following items because of their potential for PFAS contamination.**

Drinking water<br>Public waterways near waste disposal sites<br>Soils near waste disposal sites<br>Dairy products<br>Fresh produce<br>Freshwater fish<br>Seafood<br>Food packaging<br>Non-stick cookware<br>Personal hygiene products<br>Household products (fabrics, cleaning products, paints, and sealants)<br>Fire extinguishing foams<br>Fertilizers from wastewater treatment plants	☐ Will never change<br>☐ Might change<br>☐ Planning to change<br>☐ Have already changed<br>☐ Not sure

*Matrix style question
