# Supplementary material for: Let’s talk about PFAS: Inconsistent public awareness about PFAS and its sources in the United States
Source: PLoS One. 2023 Nov 16;18(11):e0294134. doi: 10.1371/journal.pone.0294134 (PMC10653490; doi:10.1371/journal.pone.0294134)
Supplement: Attachment — Submitted filename: Response to Reviewers.docx [file pone.0294134.s003.docx]

Manuscript PONE-D-23-21851

Response to Reviewers

Dear Dr. Xie,

We thank the reviewers for their constructive questions and comments on this manuscript. These suggestions have positively contributed to the quality of the manuscript. We have updated the formatting of the manuscript as requested to meet PLOS ONE’s submission requirements. Further comments from reviewers are addressed below in blue text.

Sincerely,

Stephanie DeVilleneuve

**Journal Requirements:**

1. Please ensure that your manuscript meets PLOS ONE's style requirements, including those for file naming.

**Authors’ response**: We have updated the style of the manuscript to meet PLOS ONE’s requirements.

2. Please include your full ethics statement in the ‘Methods’ section of your manuscript file. In your statement, please include the full name of the IRB or ethics committee who approved or waived your study, as well as whether or not you obtained informed written or verbal consent. If consent was waived for your study, please include this information in your statement as well.

**Authors’ response**: We have made the suggested additions to clarify IRB approval methods and consent requirements. The changes are reflected in the Methods section, lines 166-171, and reads as follows:

“The Texas A&M University Institutional Review Board (TAMU IRB) reviewed the study protocol and survey instrument prior to distribution. TAMU IRB deemed the study to be exempt from formal review. Written informed consent was obtained from all participants in the first question of the survey instrument.”

3. Please include a copy of Table 6 which you refer to in your text on page 18.

**Authors’ response:** The reference to Table 6 on page 18 was a typo. We have corrected the text to refer to Table 5.

4. Please include captions for your Supporting Information files at the end of your manuscript, and update any in-text citations to match accordingly.

**Authors’ response:** As suggested, we have added captions for the Supporting Information files and updated the in-text citations to match.

**Comments to the Author:**

Reviewer #1:

1. The online distribution of surveys could already contribute bias to represent the general US population on socioeconomics, education, and other factors. Please discuss this potential bias.

**Authors’ response:** We agree that there is general inherent bias in conducting internet research, as it excludes a portion of the population without internet access from the potential sample selection. We have added the following text in the Discussion section to acknowledge this limitation:

“Additionally, the use of an online survey instrument creates inherent bias against portions of the U.S. population who may not have internet access. An estimated 7%, or nearly 23 million Americans, do not use the internet [68]. Therefore, the sample obtained from this survey may not be generalizable to this portion of the population that is not accessible via internet-based survey instruments.”

2. One of the major finding of the article is recognized community exposure as predicting factor regarding the level of public knowledge and awareness of PFAS. Although a higher awareness of PFAS could increase information-seeking behavior, what is the application of this finding in promoting the general population awareness of PFAS?

**Authors’ response:** The application of this finding for which methods would best raise awareness of PFAS in the general population is outside of the scope of this study. Our instrument was designed to be exploratory in nature since the scientific community has no base measure of awareness within the U.S. population. The data we collected from our respondents is meant to inform the need for future work, which could focus on the determining the best way to apply this finding. We broadly discuss potential avenues for raising public awareness with support from other studies throughout the manuscript, but we feel it is outside of the scope of our data to make definitive conclusions on how best to promote PFAS awareness.

3. Detailed validation would be beneficial to look into the respondent who reported “yes” to the “Has your community been exposed to PFAS?” Are they from similar communities? Has anyone reported “no” to this question from the same communities?

**Authors’ response:** This is a good idea for a new line of inquiry with this data. However, spatial validation at this scale was not pursued in this manuscript because it is not possible to replicate multiple answers at the ZIP code scale with the sample size of 1,100 responses. Spatial analysis was not the primary goal of this study and would be better pursued in a follow-up study that addresses these details with different research questions.

Reviewer #2: In this study, Berthold et al. evaluated the general public’s awareness of PFAS contamination. They found that about half of respondents had never heard of PFAS. The respondents who are aware of PFAS community exposure were more likely to have knowledge of PFAS. They conclude that PFAS information and health risks need to be better communicated to the public to help increase awareness. This study provides a general picture of public awareness of PFAS contamination, which would have effects on social and regulatory changes in the use of PFAS. I have several questions as following:

1. The authors mentioned that the quality control checks were performed by Qualtrics. Could the authors provide detailed information about how the quality control was performed. How many surveys were received, how many surveys were used in the study. If some surveys were not used, please explain the reasons. Please provide the URL of Qualtrics used in the method part.

**Authors’ response:** Thank you for this suggestion. We have added statements to clarify what type of quality control checks Qualtrics uses. As stated in the manuscript, the total sample size was 1,100 surveys using a demographic quota to obtain a statistically representative sample of the general U.S. population. We cannot provide the number of surveys sent out in total, because Qualtrics did not provide that data. The URL of the Qualtrics survey could not be added because it is no longer active.

2. In this study, the author found that almost half of the respondents had never heard of PFAS and do not know what it is. Whether it is because the areas where these people live are not contaminated by PFAS or because they lack the education on PFAS contamination?

**Authors’ response:** This would be a good research question for a future study. What we know from our study is that people who are both unknowledgeable about PFAS and unaware of contamination in their community are unaware of PFAS overall. The survey instrument used does not delineate the cause of PFAS unawareness, therefore we believe it would be outside the scope of our study and data to attempt to address this question.

3. For the people who are aware of community exposure to PFAS, do the authors know how they learned about this information. This can help to better promote the PFAS information.

**Authors’ response:** Thank you for this suggestion. This would have been good additional data to collect. However, that information was not collected with this study and is beyond the scope of our analysis. This is a question that could be pursued in a follow-up study that specifically samples communities with exposure. Other studies that investigate sources of information in exposed communities are discussed in lines 133-135 of the Introduction. This is briefly revisited in lines 443-445 of the Discussion.

4. It is reasonable that people who are aware of PFAS exposure are more likely to know more about PFAS. As mentioned in the study, the percentage of people who are aware of PFAS exposure but have limited knowledge about PFAS was 20%. It is important for this population to learn about PFAS since they live in the area where PFAS exposure is higher. The author should discuss this point.

**Authors’ response:** This is a great point and should be included in the text. Subsequently, we added the following statement to our manuscript in lines 446-448 of the Discussion:

“However, the results also show that 20% of the respondents who are aware of PFAS exposure in their drinking water also described themselves as having limited knowledge of PFAS. Therefore, it is important to emphasize education efforts in areas where community exposure is high”.

5. In the discussion, could the author add several sentences about how to increase the public awareness about PFAS contamination in drinking water, especially in higher exposure area?

**Authors’ response:** This is not within the scope of this exploratory study. However, our intention is for this manuscript to inform future research studies focused on increasing awareness of PFAS exposure.

6. There are some typos, such as line 339 “that”.

**Authors’ response:** Thank you for pointing this out. We have corrected grammar and typos where needed.
